# Role of light transmission aggregometry in predicting diffusion-weighted imaging lesions after stent-assisted coil embolization for intracranial aneurysms: a retrospective propensity score-matched study

**DOI:** 10.3389/fneur.2026.1857176

**Published:** 2026-08-26

**Authors:** Issei Kan, Michiyasu Fuga, Toshihiro Ishibashi, Naoki Kato, Shunsuke Hataoka, Gota Nagayama, Tohru Sano, Kostadin L. Karagiozov, Yuichi Murayama

**Affiliations:** Department of Neurosugery, The Jikei University School of Medicine, Tokyo, Japan

**Keywords:** aneurysm, DWI, endovascular treatment, light transmittance aggregometry (LTA), platelet function test

## Abstract

**Object:**

The use of antiplatelet agents is essential in stent-assisted coiling (SAC) for the treatment of intracranial aneurysms, and the preoperative assessment of antiplatelet efficacy is important for reducing the risk of ischemic complications. Light transmission aggregometry (LTA) is used to assess antiplatelet drug efficacy, and we analyzed the association between patient background factors and perioperative complications in patients who underwent SAC using a retrospective analysis.

**Methods:**

Patients who underwent SAC for unruptured cerebral aneurysms from March 2017 to June 2024 were included. Dual antiplatelet therapy was administered at least 7 days before the procedure, and LTA was measured on the day of the procedure in all included patients and included in the main analysis. An additional preprocedural LTA measurement was available in 215 patients and was used to guide adjustment of antiplatelet therapy. The association between patient characteristics, ischemic complications, and postoperative diffusion-weighted imaging (DWI) positivity was evaluated. Propensity score matching, with the treatment period included as a covariate, was performed to investigate the association between adenosine diphosphate (ADP)-induced LTA values and DWI positivity.

**Results:**

A total of 1,021 unruptured aneurysms received endovascular treatment, of which 548 underwent SAC [453 Neuroform Atlas, 17 Enterprise, and 78 low-profile visualized intraluminal support (LVIS)]. The mean ADP-induced LTA value was 44.2 (range, 20–74). Symptomatic ischemic complications were present in two patients (0.4%). The overall rate of DWI-positive lesions was 52.7% (289/548). Propensity score matching with treatment period included as a covariate resulted in 152 matched pairs. Covariate balance improved after matching, with most covariates showing absolute standardized mean differences of <0.1, although some residual imbalance remained. ADP-induced LTA value was significantly higher in the DWI-positive group than in the DWI-negative group (45.89vs 43.09, *p* = 0.019).

**Conclusion:**

Higher preoperative ADP-induced LTA values were associated with postoperative DWI-positive lesions after SAC. However, given the modest discriminative performance, ADP-induced LTA values should be interpreted as a supplementary reference rather than a standalone criterion for clinical decision-making.

## Introduction

The use of antiplatelet agents is essential in stent-assisted coiling (SAC) for the treatment of intracranial aneurysms, and preoperative assessment of antiplatelet efficacy is important in reducing the risk of ischemic complications. Light transmission aggregometry (LTA) is considered the gold standard for platelet function assessment and has been used to predict the risk of ischemic complications (1). At the same time, VerifyNow, the most commonly used point-of-care platelet function testing, has increasingly been used as a simpler platelet function assessment method and is widely used to determine the efficacy of antiplatelet agents in clinical practice.

Ischemic lesions identified by DWI after endovascular treatment have been reported to occur sporadically in the postoperative period, although their clinical significance remains unclear (2–6). However, the appearance of these lesions may serve as a surrogate marker for estimating the potential risk of symptomatic thromboembolism (6–8). Various postoperative DWI-positive lesion rates have been reported, together with associated risk factors (2–6, 9–11).

To date, few reports have examined the association between ischemic lesions identified by DWI and platelet function test results, and all of them have used point-of-care testing, such as VerifyNow or TEG (5, 12, 13). Although easily applicable, these tests are also subject to variations in results depending on the timing of sample collection, and there has been no prospectively validated recommendation for therapeutic ranges (14). In some studies, discrepancies have been reported when compared with light transmission aggregometry (LTA) (15–18). Therefore, LTA was selected as the reference method in our study, as it is considered the historical gold standard.

In this study, we analyzed the association between patient background factors, including preoperative LTA values, and perioperative complications, including MRI changes (postoperative DWI-positive lesions) in patients who underwent SAC using a retrospective analysis.

## Methods

### Study population

**Figure 1:**
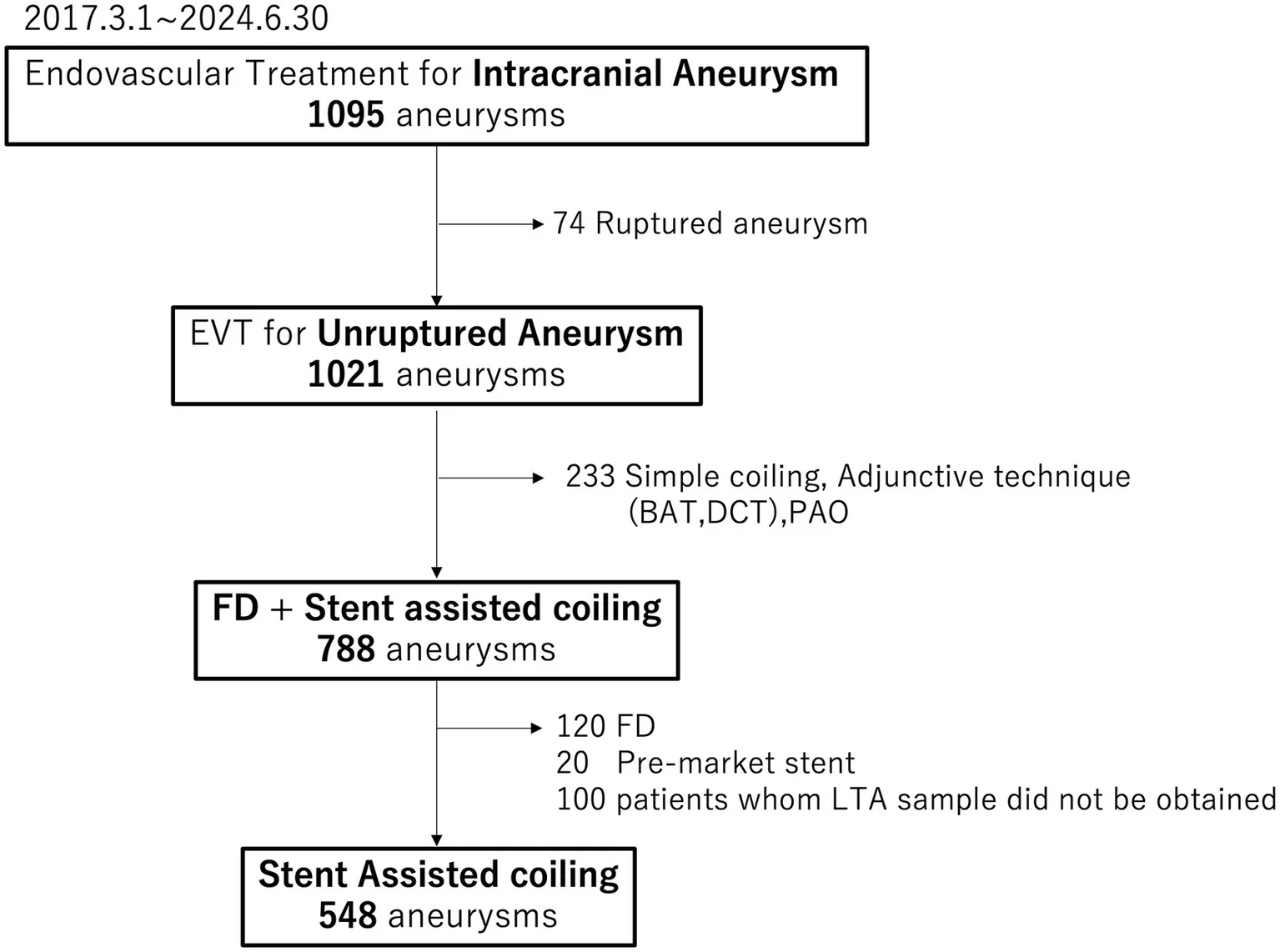
Flow chart showing patient inclusion into the present study. BAT: balloon assisted technique; DCT: double catheter technique; PAO: parent artery occlusion; FD: flow diverter; LTA: light transmission aggregometry.

Consecutive patients with UIAs receiving a combined aspirin and clopidogrel protocol from 1 March 2017 to 30 June 2024 were retrospectively reviewed. Dual antiplatelet therapy (DAPT) was administered as the standard protocol for all patients with UIAs for at least 7 days before endovascular surgery at our institution from July 2015. Exclusion criteria were simple coiling, parent artery occlusion, flow-diverter stent placement, and patients without LTA testing (Figure 1).

Baseline information, such as age, sex, alcohol consumption, smoking, family history, medical history, aneurysm shape, multiple aneurysms, symptomatic aneurysms, maximum diameter, neck diameter, aneurysm site, left or right side, first treatment or not, stent type, number of stents, and clopidogrel or prasugrel use, was collected by reviewing medical and radiological records. As the data obtained were from routine examinations, which were consented to in full by each patient on admission and were applied to retrospective analysis, specific informed consent for this study was waived.

This study was approved by the Institutional Review Board and ethics committee (Nos. 26-050 and 7,555), and the need for patient consent was waived based on its retrospective design.

### Endovascular procedure and MRI protocol

Two antiplatelet drugs (aspirin 100 mg and either clopidogrel 75 mg or prasugrel 3.75 mg) were administered at least 7 days prior to the procedure. LTA was measured on the day of the procedure in all included patients and was used for the main analysis. An additional preprocedural LTA measurement was obtained from 215 patients, with a mean interval of 1.96 days before the procedure, to guide antiplatelet medication adjustment. For patients with a preprocedural ADP-induced LTA value >60, the antiplatelet regimen was adjusted according to the protocol described below. LTA was performed using the PA200C system (Kyowa Hakko Bio Co., Tokyo, Japan). Clopidogrel hyporesponsiveness was defined as a maximum platelet aggregation of >60% in response to 5 μmol/L ADP stimulation. The detailed protocol was as follows: If the preoperative LTA value was >60, additional clopidogrel 75 mg (boost) was administered before August 2020, patients receiving prasugrel 3.75 mg or 2.5 mg underwent switching, and the adjusted antiplatelet regimen was continued until the day of surgery.

All stent coiling embolization procedures were accomplished under general anesthesia in a standardized fashion and were conducted exclusively by or under the supervision of certified interventional neurosurgeons. After sheath insertion, 4,000–5,000 U of heparin was given intravenously as a bolus infusion, followed by 1,000–2,000 U of heparin intermittently to maintain the activated clotting time during the procedure at a level of 250–300 s. The embolization was conducted as follows: The femoral artery was selected as the access site, and for anterior circulation aneurysms, either a 6-F guiding sheath or an 8-F balloon-guiding catheter was placed in the cervical portion of the internal carotid artery, followed by the advancement of microcatheters for stent deployment and coil delivery. All patients underwent head MRI (MAGNETOM Skyra 3.0 T, Siemens Healthcare GmbH, Forchheim, Germany) within 48 h after the procedure.

### Assessment

**Figure 2:**
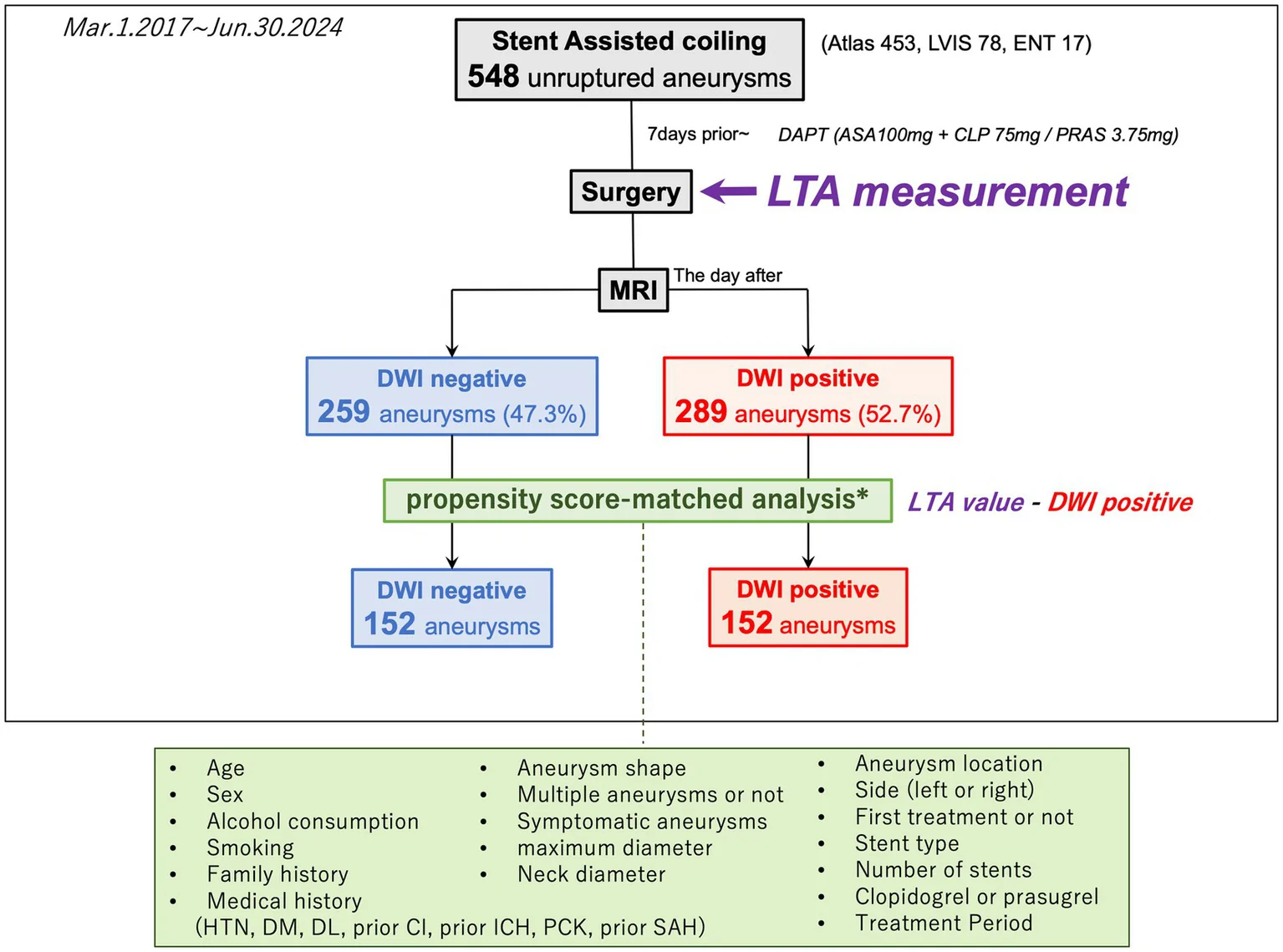
Schema of this study after patient enrollment. DAPT: dual antiplatelet therapy, ASA: aspirin; CLP: clopidogrel, PRAS: prasugrel; DWI: diffusion weighted image.

The association between each patient’s background factors and ischemic complications during the hospitalization period—defined as one or more grades of deterioration in the mRS—and postoperative DWI positivity was analyzed. DWI-positive lesions were defined as *de novo* hyperintense areas on diffusion-weighted imaging, regardless of whether they were symptomatic or asymptomatic. All postoperative DWI images were independently assessed by two interventional neurosurgeons. In cases of disagreement between the two investigators, a third interventional neurosurgeon participated in the evaluation, and the final DWI classification was determined by consensus. To investigate whether LTA value affected the DWI-positive rate, propensity score-matched analysis was used (Figure 2).

### Statistical analysis

For the comparison of baseline characteristics between DWI-negative and DWI-positive groups, continuous variables were expressed as the mean (standard deviation) and compared using Student’s t-test. Categorical variables were expressed as the number (percentage) and compared using Fisher’s exact test. A 1:1 propensity score matching between the DWI-negative and DWI-positive groups was performed using the nearest neighbor method without replacement and with a caliper width of 0.20, to match groups based on the covariates of age, sex, alcohol consumption, smoking status, family history, medical history, aneurysm shape, multiple aneurysms, symptomatic aneurysms, maximum diameter, neck diameter, aneurysm site, left or right side, first treatment or not, stent type, number of stents, clopidogrel or prasugrel use, and treatment period. To account for the change in the antiplatelet adjustment protocol, treatment period was defined as before August 2020 and August 2020 or later. Covariate balance before and after propensity score matching was assessed using standardized mean differences. An absolute standardized mean difference of <0.1 was considered to indicate adequate balance. There were no missing data for the variables included in the propensity score-matching analysis. Receiver operating characteristic curve analysis was performed to evaluate the ability of ADP-induced LTA values to discriminate between postoperative DWI positivity and negativity. The optimal cutoff value was determined using the Youden index. The area under the curve was calculated with a 95% confidence interval. All statistical analyses were performed using R and R Commander-based Easy R (EZR) software (Saitama Medical Center, Jichi Medical School, Saitama, Japan) (19). *p*-values < 0.05 were considered statistically significant.

## Results

### Study population and characteristics

During the observation period, 1,021 unruptured cerebral aneurysms received endovascular treatment at our department, of which 548 underwent coil embolization with stenting. The mean (SD) aneurysm diameter and neck diameter were 6.22 mm (2.5) and 5.18 mm (2.1), respectively. The type of stent and the actual number of stents used were 453 for Atlas stents, 17 Enterprise stents, and 78 LVIS stents, respectively (Table 1).

**Table 1:** Patient demographics.

Characteristics	*n* = 548
Sex (%)	
Female	395 (72)
Age (mean (SD))	58.7 (12.2)
Medical history	
Hypertension (%)	214 (39.1)
Diabetes mellitus (%)	26 (4.7)
Dyslipidemia (%)	103 (18.8)
Prior cerebral infarction (%)	10 (1.8)
Prior cerebral hemorrhage(%)	1 (0.18)
Poly cystic kidney disease(%)	15 (2.7)
Past history of SAH (%)	8 (1.5)
Family history	
Family.history. PKD (%)	5 (0.9)
Family.history. SAH (%)	86(15.7)
Family.history. UIA (%)	19 (3.4)
Smoking (%)	
Current	63 (11.4)
None	343 (62.6)
Past smoker	142 (25.9)
Drinking (%)	189 (34.5)
Treatment period (*N* (%))	
Early period	215 (39)
Late period	333 (61)
Aneurysm characterics	
Maximum diameter (mean (SD))	6.22 (2.5)
Neck.mm (mean (SD))	5.18 (2.1)
Bleb formation(%)	146 (26.6)
Symptomatic aneurysm (%)	5 (0.9)
Location (%)	
ACA/ACom	92 (16.8)
BA/SCA	46 (8.4)
ICA	316 (57.7)
MCA	44 (8.0)
PCA	5 (0.9)
VA/PICA	45 (8.2)
Form (%)	
Dissecting	17 (3.1)
Fusiform	10 (1.8)
Saccular	521 (95.1)
Treatment (%)	
Initial treatment	466(85)
Retreatment	82 (15)
Side left (%)	286 (52)
Multipe aneurysm (%)	150 (27.4)
Variety of stent (%)	
Atlas	453 (82.7)
Enterprise	17 (3.1)
LVIS	78 (14.2)
Number of Stent use (%)	
1	542 (98.9)
2	6 (1.1)
Treatment outcome	
Raymond.scale (%)	
Class1	131 (23.9)
Class 2	276 (50.4)
Class 3	141 (25.7)
VER. Mean (SD)	28.4 (6.8)
Symptomatic Ischemic complication (%)	2 (0.4)
ADP inhibitor (%)	
Clopidogrel	453 (82.7)
Prasugrel	95 (17.3)
LTA. ADP. value (mean (SD))	44.1 (10.3)
LTA. collagen. Value (mean (SD))	47.4 (12.8)

### APT management and LTA value

The mean (SD) ADP-induced and collagen-induced LTA values on the day of the procedure were 44.1 (10.3) and 47.4 (12.8), respectively. Preprocedural LTA measurements were available in 215 patients and were obtained at a mean of 1.96 days before the procedure. Among the 31 patients with a preprocedural ADP-induced LTA value >60, 14 received an additional dose of clopidogrel, and 17 were switched to prasugrel. On the day of the procedure, 26 patients had achieved an LTA value ≤60, whereas 5 remained with values >60. All five patients with persistent LTA values >60 were in the clopidogrel-boost group. In the entire cohort, 37 of 548 patients had a day-of-procedure LTA value >60. No additional intervention was performed solely on the basis of the day-of-procedure LTA result.

### Rate of ischemic complications and DWI-positive lesions

Symptomatic ischemic complications were present in two patients (0.4%). The rate of postoperative DWI-positive lesions was 52.7% (289/548). There were no hemorrhagic complications during the observation period.

### Factors associated with DWI-positive lesions

**Figure 3:**
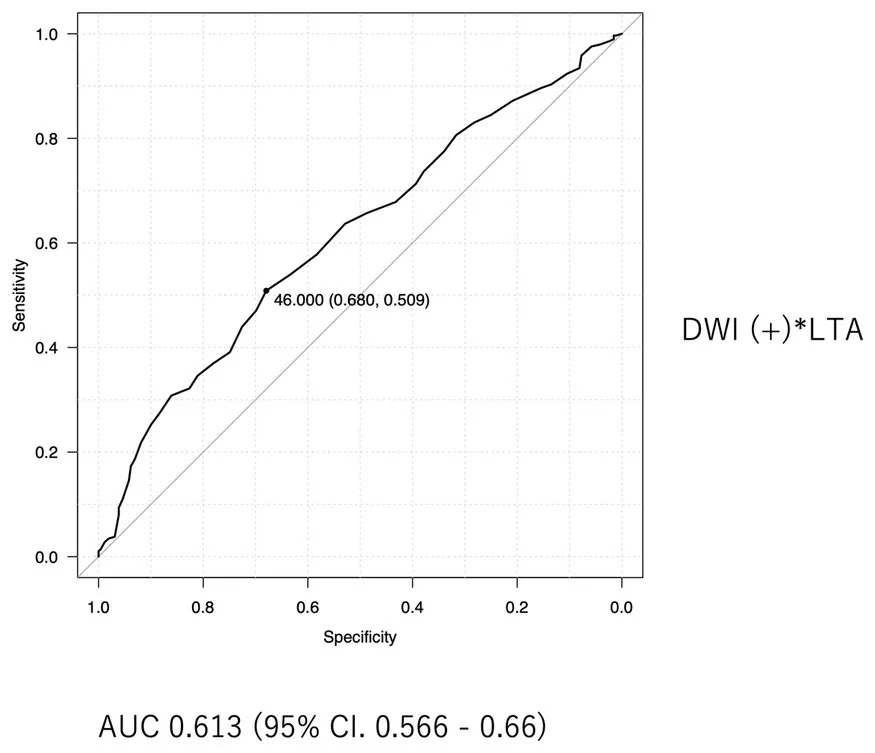
Receiver operating characteristic curve analysis of relations between DWI positivity related and ADP-induced LTA. DWI: diffusion weighted image.

Propensity score matching, including treatment period as a covariate, resulted in 152 matched pairs in the DWI-positive and DWI-negative groups, and the results are presented in Table 2. Covariate balance improved after matching, with most covariates showing absolute standardized mean differences <0.1, although residual imbalance remained for some covariates. In the matched cohort, the difference in collagen-induced aggregation values between the DWI-positive and DWI-negative groups was no longer statistically significant. In contrast, ADP-induced LTA values remained significantly higher in the DWI-positive group than in the DWI-negative group (45.89 vs. 43.09, *p* = 0.019). Receiver operating characteristic curve analysis identified an ADP-induced LTA cutoff value of 46.0 for postoperative DWI positivity, with an area under the curve of 0.613 (95% CI, 0.566–0.660) (Figure 3).

**Table 2:** Clinical, anatomical, and procedural characteristics and outcomes according to postoperative DWI-positive lesions before and after propensity score matching.

Variable	Total population				Propensity score matching			
	DWI negative (*n* = 259)	DWI positive (*n* = 289)	*p*-value	SMD	DWI negative (*n* = 152)	DWI positive (*n* = 152)	*p*-value	SMD
Sex, female (%)	184 (71.0)	211 (73.0)	0.634	0.044	112 (73.7)	116 (76.3)	0.691	0.061
Age (mean (SD))	57.48 (12.04)	59.99 (12.40)	0.017	0.205	58.79 (11.61)	58.55 (12.30)	0.859	0.02
Medical history								
Hypertension (%)	91 (35.1)	123 (42.6)	0.08	0.153	56 (36.8)	58 (38.2)	0.906	0.027
Diabetes Mellitus (%)	11 (4.2)	15 (5.2)	0.689	0.044	5 (3.3)	7 (4.6)	0.77	0.068
Dyslipidemia (%)	52 (20.1)	51 (17.6)	0.512	0.062	30 (19.7)	26 (17.1)	0.657	0.068
Prior cerebral infarction (%)	2 (0.8)	8 (2.8)	0.111	0.152	1 (0.7)	1 (0.7)	1	<0.001
Prior cerebral hemorrhage(%)	1 (0.4)	0 (0.0)	0.473	0.088	0 (0.0)	0 (0.0)	1	<0.001
Poly cystic Kidney disease(%)	3 (1.2)	12 (4.2)	0.037	0.187	1 (0.7)	2 (1.3)	1	0.067
Past history of SAH (%)	1 (0.4)	7 (2.4)	0.073	0.173	1 (0.7)	1 (0.7)	1	<0.001
Family history								
Family.history. PKD (%)	3 (1.2)	2 (0.7)	0.671	0.049	0 (0.0)	2 (1.3)	0.498	0.163
Family.history. SAH (%)	40 (15.4)	46 (15.9)	0.907	0.013	26 (17.1)	24 (15.8)	0.877	0.035
Family.history. UIA (%)	5 (1.9)	14 (4.8)	0.099	0.162	3 (2.0)	4 (2.6)	1	0.044
Smoking								
Current	31 (12.0)	32 (11.1)	0.538	0.094	18 (11.8)	17 (11.2)	0.831	0.07
None	156 (60.2)	187 (64.7)			95 (62.5)	100 (65.8)		
Past smoker	72 (27.8)	70 (24.2)			39 (25.7)	35 (23.0)		
Drinking (%)	91 (35.1)	98 (33.9)	0.788	0.026	50 (32.9)	44 (28.9)	0.535	0.085
Treatment period (%)								
Early period	70 (27.0)	145 (50.2)	<0.001	0.489	66 (43.4)	66 (43.4)	1	<0.001
Late period	189 (73.0)	144 (49.8)			86 (56.6)	86 (56.6)		
Aneurysm characterics								
Maximum diameter (mean (SD))	5.91 (2.28)	6.50 (2.82)	0.008	0.231	6.10 (2.54)	6.12 (1.84)	0.956	0.006
Neck.mm (mean (SD))	4.87 (1.68)	5.45 (2.44)	0.002	0.274	5.05 (1.90)	5.13 (1.55)	0.699	0.044
Bleb formation(%)	68 (26.3)	78 (27.0)	0.923	0.017	40 (26.3)	42 (27.6)	0.897	0.03
Symptomatic aneurysm (%)	0 (0.0)	5 (1.7)	0.063	0.188	0 (0.0)	0 (0.0)	1	<0.001
Location (%)								
ACA/ACom	28 (10.8)	64 (22.1)	NA	0.354	22 (14.5)	12 (7.9)	0.516	0.236
BA/SCA	19 (7.3)	27 (9.3)			13 (8.6)	14 (9.2)		
ICA	169 (65.3)	147 (50.9)			92 (60.5)	101 (66.4)		
MCA	19 (7.3)	25 (8.7)			13 (8.6)	10 (6.6)		
PCA	2 (0.8)	3 (1.0)			2 (1.3)	2 (1.3)		
VA/PICA	22 (8.5)	23 (8.0)			10 (6.6)	13 (8.6)		
Form (%)								
Dissecting	8 (3.1)	9 (3.1)	0.745	0.069	5 (3.3)	5 (3.3)	1	<0.001
Fusiform	6 (2.3)	4 (1.4)			4 (2.6)	4 (2.6)		
Saccular	245 (94.6)	276 (95.5)			143 (94.1)	143 (94.1)		
Treatment								
Initinal Treatment (%)	227 (87.6)	239 (82.7)	0.119	0.14	133 (87.5)	134 (88.2)	1	0.02
Retreatment(%)	32 (12.4)	50 (17.3)			19 (12.5)	18 (11.8)		
Side, left(%)	140 (54.1)	146 (50.5)	0.441	0.071	79 (52.0)	80 (52.6)	1	0.013
Multipe aneurysm (%)	67 (25.9)	83 (28.7)	0.502	0.064	42 (27.6)	45 (29.6)	0.8	0.044
Stent type								
Atlas	198 (76.4)	255 (88.2)	<0.001	0.362	130 (85.5)	131 (86.2)	0.854	0.084
Enterprise	7 (2.7)	10 (3.5)			5 (3.3)	3 (2.0)		
LVIS	54 (20.8)	24 (8.3)			17 (11.2)	18 (11.8)		
Number of Stent use (%)								
1	258 (99.6)	284 (98.3)	0.22	0.132	151 (99.3)	151 (99.3)	1	<0.001
2	1 (0.4)	5 (1.7)			1 (0.7)	1 (0.7)		
Raymond.scale (%)								
Class 2	130 (50.2)	146 (50.5)	0.161	0.164	84 (55.3)	81 (53.3)	0.705	0.093
Class 3	59 (22.8)	82 (28.4)			37 (24.3)	43 (28.3)		
Class1	70 (27.0)	61 (21.1)			31 (20.4)	28 (18.4)		
VER. (mean (SD))	29.23 (6.64)	27.64 (6.85)	0.012	0.236	28.54 (6.99)	27.93 (6.91)	0.481	0.087
Symptomatic Ischemic complication (%)^†^	0 (0.0)	2 (0.7)	0.501	0.118	0 (0.0)	2 (1.3)	0.498	0.163
ADP inhibitor								
Clopidogrel	201 (77.6)	252 (87.2)	0.003	0.254	128 (84.2)	131 (86.2)	0.747	0.056
Prasugrel	58 (22.4)	37 (12.8)			24 (15.8)	21 (13.8)		
LTA								
ADP. value (mean (SD))	42.01 (9.66)	46.12 (10.59)	<0.001	0.406	43.09 (9.98)	45.89 (10.68)	0.019	0.271
collagen. Value (mean (SD))	45.75 (12.43)	48.89 (12.92)	0.006	0.248	45.43 (12.85)	47.85 (12.93)	0.11	0.188

ICA, internal carotid artery; NA, not available; RROC, Raymond–Roy Occlusion Classification; SMD, standardized mean difference; VER, volume embolization ratio. ^†^Symptomatic complications were defined as an increase of ≥1 point in the modified Rankin Scale score compared with the preoperative level. *p < 0.05. Unless otherwise indicated, values are presented as the number (%) or mean (standard deviation). Percentages may not total 100 because of rounding.

## Discussion

The correlation between the presence of postoperative DWI-positive lesions and patient background factors after stent coiling was evaluated. Preoperative ADP-induced LTA test values were significantly higher in the DWI-positive lesion group compared with the negative group. Propensity score-matched analysis also showed that only the preoperative ADP-induced LTA value was a significantly associated factor.

A limited number of papers have shown a correlation between DWI-positive lesions and the effect of antiplatelet agents evaluated with TEG or VerifyNow (5, 12, 13), and the current study is the first to report such a correlation using LTA.

### Postoperative MR evaluation

In this study, the DWI positivity rate was 52%, which was within the established ranges after stent-assisted coiling (3, 9–11, 20). We considered DWI-positive lesions as those that could be confirmed by two interventional neurosurgeons. Some reports have examined the relationship between the number of DWI-positive lesions and symptomatic cerebral infarction (7, 8). Further studies on the number, size, and location of DWI-positive lesions may help to elucidate this relationship in more detail.

### Correlation of different platelet function test results with DWI changes

Many studies have reported postoperative DWI-positive rates in relation to multiple clinical and procedural factors. However, only a limited number of studies have evaluated the association between platelet function test results and postoperative DWI-positive lesions. These studies have used point-of-care assays, such as VerifyNow and thromboelastography (4, 5, 13). Another report examined the association between imaging findings and high platelet reactivity after antiplatelet treatment adjustment, but neither the DWI-positive rate nor the LTA values were reported (21). To our knowledge, this study is the first to demonstrate an association between specific ADP-induced LTA values and postoperative DWI-positive lesions after SAC. The ROC analysis identified an ADP-induced LTA cutoff value of 46.0 for postoperative DWI positivity; however, the AUC was 0.613, indicating only modest discriminative ability. Therefore, this cutoff should not be interpreted as a definitive clinical threshold. Rather, ADP-induced LTA values may serve as a supplementary reference for antiplatelet management when considered together with other clinical and procedural factors.

Moreover, CYP2C19 genotyping can identify patients with reduced responsiveness to clopidogrel and may help guide individualized antiplatelet therapy (22). However, it was not routinely incorporated into our institutional practice during the study period because of limited insurance reimbursement for routine use in this clinical setting in our country and the need for testing at an external laboratory. Therefore, CYP2C19 genotyping was not performed in this study.

### Stent type and DWI positivity

The DWI-positive rates for Enterprise, Neuroform Atlas, and LVIS stents were 58.8, 56.3, and 30.7%, respectively. The corresponding mean ADP-induced LTA values on the day of surgery were 48, 44, and 42, respectively (Table 3). Although the DWI-positive rates and mean ADP-induced LTA values showed similar numerical trends among stent types, stent type was not significantly associated with DWI positivity after propensity score matching. As medication adherence and relevant procedural characteristics were not systematically evaluated, the observed differences among stent types should be interpreted cautiously.

**Table 3:** Stent type and DWI positivity.

Variables	*n*	DWI positive number	DWI positive rate (%)	Mean ADP
LVIS	78	24	30.77	42.6
Atlas	453	255	56.29	44.3
Enterprise	17	10	58.82	48.4

### DWI-positive lesion

Air embolism, coating material from endovascular devices, and atherosclerotic plaques have been reported as potential causes of postoperative DWI-positive lesions (23–26). Moreover, DWI positivity has been associated with symptomatic cerebral infarction and cognitive decline (27–30). Propensity score matching reduced measured baseline imbalance between the DWI-positive and DWI-negative groups; however, residual confounding from unmeasured atherosclerotic and procedural factors cannot be excluded. In particular, the number of catheters, use of intermediate catheters, and the number of coils were not systematically evaluated. Further studies incorporating these procedural characteristics are warranted.

### LTA and aggregation suppression in stent coiling

In this study, symptomatic ischemic stroke occurred in 0.4% of patients (2/548). This low event rate limited our ability to evaluate factors associated with symptomatic ischemic complications (31).

Switching from clopidogrel to prasugrel in patients with high on-treatment platelet reactivity may have resulted in platelet inhibition (32–35). However, antiplatelet medication on the day of surgery did not differ significantly between the DWI-positive and DWI-negative groups after propensity score matching. As the antiplatelet-adjustment protocol changed during the study period, the treatment period was considered a potential confounder and was incorporated into the propensity score-matching model. After the treatment period was incorporated into the model, ADP-induced LTA values remained significantly associated with postoperative DWI-positive lesions. This suggests that the observed association was not solely attributable to the protocol change. Nevertheless, residual temporal confounding cannot be completely excluded because this was a retrospective, single-center study, and treatment strategies evolved over time. Furthermore, evidence regarding low-dose prasugrel remains limited, and further studies are warranted (34).

As reported previously (31), an LTA value of 60% has been used as a target to reduce the risk of perioperative ischemic complications. No hemorrhagic complications were observed under the current protocol; however, the present study was not designed to establish the safety of a specific LTA target.

### Limitations

This study has several limitations. First, it was a retrospective, single-center study and did not include patients treated with flow-diverter devices; therefore, the generalizability of our findings may be limited. Second, procedural techniques and treatment strategies evolved during the study period, including changes in antiplatelet management and the use of intermediate catheters. Although the treatment period was included in the propensity score-matching model, residual temporal and procedural confounding cannot be completely excluded. Although propensity score matching improved covariate balance, residual imbalance remained for some covariates and may have influenced the results. Third, baseline LTA values before the initiation of antiplatelet therapy were not measured. In addition, CYP2C19 genotyping was not routinely incorporated into our institutional antiplatelet management protocol; therefore, the potential influence of genetic variation in clopidogrel metabolism could not be evaluated. Fourth, postoperative DWI findings were analyzed only as a binary outcome. The number, size, anatomical distribution, and lesion-level clinical relevance of DWI-positive lesions were not evaluated. Postoperative cognitive function was also not systematically assessed; therefore, the relationship between DWI-positive lesions and long-term cognitive outcomes could not be evaluated. Finally, because the initial independent DWI ratings were not preserved in a structured dataset, Cohen’s kappa coefficient could not be calculated retrospectively, and inter-rater agreement could not be quantitatively assessed.

## Conclusion

Higher preoperative ADP-induced LTA values were associated with postoperative DWI positivity after SAC. However, given the modest discriminative performance, ADP-induced LTA values should be interpreted as a supplementary reference rather than as a standalone threshold for clinical decision-making.

## Data Availability

The raw data supporting the conclusions of this article will be made available by the authors, without undue reservation.

## Supplementary material

SUPPLEMENTARY Figure S1 Love plot showing standardized mean differences before and after propensity score matching. The Love plot shows the absolute standardized mean differences for each covariate before and after propensity score matching. The vertical dashed line indicates an absolute standardized mean difference of 0.1.

SUPPLEMENTARY Figure S2 Visual abstract of this study.
